# 2,4,6,8-Tetra­kis(4-chloro­phen­yl)-3,7-diaza­bicyclo­[3.3.1]nonan-9-one *O*-benzyl­oxime acetone monosolvate

**DOI:** 10.1107/S1600536812016509

**Published:** 2012-04-21

**Authors:** Dong Ho Park, V. Ramkumar, P. Parthiban

**Affiliations:** a Department of Biomedicinal Chemistry, Inje University, Gimhae, Gyeongnam 621 749, Republic of Korea; b Department of Chemistry, IIT Madras, Chennai 600 036, TamilNadu, India

## Abstract

In the title compound, C_38_H_31_Cl_4_N_3_O·C_3_H_6_O, the 3,7-diaza-bicycle exists in a chair–boat conformation. The 4-chloro­phenyl groups attached to the chair form are equatorially oriented at an angle of 18.15 (3)° with respect to each other, whereas the 4-chloro­phenyl groups attached to the boat form are oriented at an angle of 32.64 (3)°. In the crystal, mol­ecules are linked by N—H⋯π and C—H⋯O inter­actions.

## Related literature

For the synthesis and stereochemistry of 3,7-diaza­bicyclo­[3.3.1]nonan-9-one derivatives, see: Parthiban *et al.* (2008 ▶). For the biological activity of 3,7-diaza­bicyclo­[3.3.1]nonan-9-one derivatives and related structures, see: Parthiban *et al.* (2009 ▶, 2010 ▶); Asakawa (1995 ▶); Jayaraman & Avila (1981 ▶). For ring puckering parameters, see: Cremer & Pople (1975 ▶); Nardelli (1983 ▶); Luger & Bülow (1983 ▶).
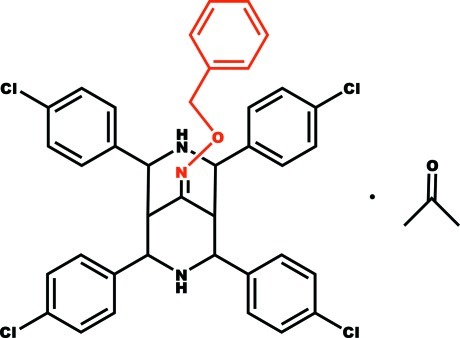


## Experimental

### 

#### Crystal data

C_38_H_31_Cl_4_N_3_O·C_3_H_6_O
*M* *_r_* = 745.54
Monoclinic, 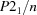

*a* = 14.9237 (5) Å
*b* = 10.5064 (3) Å
*c* = 24.6015 (7) Å
β = 93.116 (1)°
*V* = 3851.7 (2) Å^3^
*Z* = 4
Mo *K*α radiation
μ = 0.35 mm^−1^
*T* = 293 K
0.20 × 0.16 × 0.16 mm

#### Data collection

Bruker APEXII CCD diffractometer
Absorption correction: multi-scan (*SADABS*; Bruker, 2004 ▶) *T*_min_ = 0.934, *T*_max_ = 0.947
35931 measured reflections
7173 independent reflections
4263 reflections with *I* > 2σ(*I*)
*R*_int_ = 0.040

#### Refinement

*R*[*F* ^2^ > 2σ(*F* ^2^)] = 0.062
*wR*(*F* ^2^) = 0.188
*S* = 1.03
7173 reflections
475 parameters
H atoms treated by a mixture of independent and constrained refinement
Δρ_max_ = 0.89 e Å^−3^
Δρ_min_ = −0.29 e Å^−3^

### 

Data collection: *APEX2* (Bruker, 2004 ▶); cell refinement: *SAINT-Plus* (Bruker, 2004 ▶); data reduction: *SAINT-Plus*; program(s) used to solve structure: *SIR92* (Altomare *et al.*, 1993) ▶; program(s) used to refine structure: *SHELXL97* (Sheldrick, 2008 ▶); molecular graphics: *ORTEP-3* (Farrugia, 1997 ▶); software used to prepare material for publication: *SHELXL97*.

## Supplementary Material

Crystal structure: contains datablock(s) global, I. DOI: 10.1107/S1600536812016509/hb6689sup1.cif

Structure factors: contains datablock(s) I. DOI: 10.1107/S1600536812016509/hb6689Isup2.hkl

Supplementary material file. DOI: 10.1107/S1600536812016509/hb6689Isup3.cml

Additional supplementary materials:  crystallographic information; 3D view; checkCIF report

## Figures and Tables

**Table 1:** Hydrogen-bond geometry (Å, °) *Cg*3 is the centroid of the C8–C13 ring.

*D*—H⋯*A*	*D*—H	H⋯*A*	*D*⋯*A*	*D*—H⋯*A*
C13—H13⋯O2	0.93	2.52	3.441 (6)	172
N2—H2*A*⋯*Cg*3^i^	0.88 (3)	2.85 (3)	3.637 (3)	150 (3)

Symmetry code: (i) 
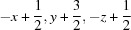
.

**Fig. 1.**
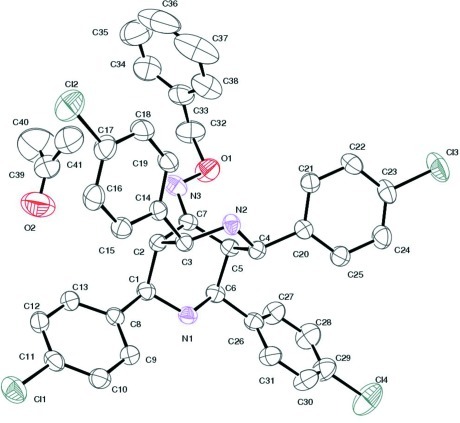
Anistropic displacement representation of the molecule with atoms represented with 30% probability ellipsoids (H atoms are removed for clarity).

## supplementary crystallographic information

### Comment

The 3,7-diazabicyclo[3.3.1] nonan-9-one nucleus is widely present in Lupin alkaloids and are displaying various biological actions (Parthiban *et al.*, 2009, 2010, Asakawa, 1995, Jayaraman & Avila, 1981). In fact, the biological activities mainly depends on the nature of the substituents and their positions on the nucleus. Since C=N—O-*R* is an important class of pharmacophore by displaying biological actions, we have synthesized the title molcule by the condensation of 3,7-diazabicyclo[3.3.1]nonan-9-one and *O*-benzyl moiety to make the biologically potent oxime derivative. Because the biological actions mainly depend on the stereochemistry of the molecule, we undertaken the title molecule for the present study to explore its stereochemistry.

The crystallographic parameters *viz*., torsion angles, asymmetry parameters and ring puckering parameters calculated for the title compound shows that one of the piperidone rings, N(1)—C(1)—C(2)—C(7)—C(5)—C(6) adopts a near ideal chair conformation, according to Cremer & Pople and Nardelli. The total puckering amplitude, Q_T_ is 0.606 (3) Å, the phase angle θ is 2.7 (3)° and phi is 261 (6)° (Cremer & Pople, 1975). The smallest displacement asymmetry parameters q_2_ and q_3_ are 0.031 (3) and 0.605 (3) Å, respectively (Nardelli, 1983). On the otherhand, another piperidone ring N(2)—C(3)—C(2)—C(7)—C(5)—C(4) exists in the boat conformation according to C&P by Q_T_ = 0.764 (3), θ = 91.9 (2)° and phi = 357.0 (3)° as well as Nardelli by q_2_ = 0.764 (3) and q_3_ = -0.025 (3)°.

An equatorial orientation of the 4-chlorophenyl groups attached on the chair form piperidone is supported by the angles of C(1)—C(8) and C(6)—C(26) on the C&P plane normal as 70.85 (19) and 75.04 (19)°, respectively (Luger & Bülow, 1983). The equatorial orientations of the aryl groups are further supported by thier torsion angles; the C8—C1—C2—C7 is 178.4 (3)° and C26—C6—C5—C7 is 177.0 (3)°. The 4-chlorophenyl groups attached on the boat form of the piperidone have angles with C&P plane normal are C(3)—C(14) = 57.34 (19)° and C(4)—C(20) = 61.70 (19)°, they are respectively in bisectional and equatorial orientations according to Luger & Bulow. In fact, both lies on the boundary of bisectional and this is further supported by their torsion angles as follows: C7—C2—C3—C14 = 115.5 (3)° and C20—C4—C5—C7 = -121.6 (3)°.

The 4-chlorophenyl groups attached on the chair form are equatorially oriented at an angle of 18.15°, respect to each other, whereas, the 4-chlorophenyl groups attached to the boat form are oriented at an angle of 32.64° between them.

Based on the above analysis, it is clear that the title compound exists in the chair-boat conformation with an equatorial orientation of the 4-chlorophenyl groups on both sides of the secondary amino group of the piperidone in the chair conformation.

The crystal packing is stabilized by weak intermolecular N—H···π (C14—C19) and C—H···O interactions.

### Experimental

The 2,4,6,8-*tetrakis*(4-chlorophenyl)-3,7-diazabicyclo[3.3.1]nonan-9-one was synthesized by a modified Mannich condensation in one-pot, using 4-chlorobenzaldehyde (0.2 mol, 28.12 g), acetone (0.05 mol, 3.7 ml) and ammonium acetate (0.1 mol, 7.7 g) in a 50 ml of absolute ethanol (Parthiban *et al.*, 2008). The mixture was gently warmed on a hot plate at 303 K (30° C) with moderate stirring till the complete consumption of the starting materials, which was monitored by TLC. At the end, the crude 3,7-diazabicycle was separated by filtration and gently washed with 1:5 cold ethanol-ether mixture. The pure 2,4,6,8-*tetrakis*(4-chlorophenyl)-3,7-diazabicyclo[3.3.1]nonan-9-one (0.01 mol, 5.823 g) was condensed with *O*-benzylhydroxylamine hydrochloride (0.012 mol, 1.915 g) using sodium acetate trihydrate (0.03 mol, 3.06 g) as base in ethanol-chloroform 1:1 mixture to obtain the title oxime ether. Colourless prisms were obtained by slow evaporation of an acetone solution.

### Refinement

The nitrogen H atom was located in a difference Fourier map and refined isotropically. Other hydrogen atoms were fixed geometrically and allowed to ride on the parent carbon atoms with aromatic C—H = 0.93 Å, aliphatic C—H = 0.98 Å and methylene C—H = 0.97 Å. The displacement parameters were set for phenyl, methylene and aliphatic H atoms at *U*_iso_(H) = 1.2*U*_eq_(C) and for methyl H atoms at *U*_iso_(H) = 1.5*U*_eq_(C).

### Crystal data

**Table:**

C_38_H_31_Cl_4_N_3_O·C_3_H_6_O	*F*(000) = 1552
*M**_r_* = 745.54	*D*_x_ = 1.286 Mg m^−^^3^
Monoclinic, *P*2_1_/*n*	Mo *K*α radiation, λ = 0.71073 Å
Hall symbol: -P 2yn	Cell parameters from 6628 reflections
*a* = 14.9237 (5) Å	θ = 1.6–25.0°
*b* = 10.5064 (3) Å	µ = 0.35 mm^−^^1^
*c* = 24.6015 (7) Å	*T* = 293 K
β = 93.116 (1)°	Prism, colourless
*V* = 3851.7 (2) Å^3^	0.20 × 0.16 × 0.16 mm
*Z* = 4	

### Data collection

**Table:**

Bruker APEXII CCD diffractometer	7173 independent reflections
Radiation source: fine-focus sealed tube	4263 reflections with *I* > 2σ(*I*)
Graphite monochromator	*R*_int_ = 0.040
ω and φ scan	θ_max_ = 25.5°, θ_min_ = 1.6°
Absorption correction: multi-scan (*SADABS*; Bruker, 2004)	*h* = −18→18
*T*_min_ = 0.934, *T*_max_ = 0.947	*k* = −12→8
35931 measured reflections	*l* = −29→29

### Refinement

**Table:**

Refinement on *F*^2^	Primary atom site location: structure-invariant direct methods
Least-squares matrix: full	Secondary atom site location: difference Fourier map
*R*[*F*^2^ > 2σ(*F*^2^)] = 0.062	Hydrogen site location: inferred from neighbouring sites
*wR*(*F*^2^) = 0.188	H atoms treated by a mixture of independent and constrained refinement
*S* = 1.03	*w* = 1/[σ^2^(*F*_o_^2^) + (0.0699*P*)^2^ + 3.3824*P*] where *P* = (*F*_o_^2^ + 2*F*_c_^2^)/3
7173 reflections	(Δ/σ)_max_ = 0.005
475 parameters	Δρ_max_ = 0.89 e Å^−^^3^
0 restraints	Δρ_min_ = −0.29 e Å^−^^3^

### Special details

**Table:**

Geometry. All e.s.d.'s (except the e.s.d. in the dihedral angle between two l.s. planes) are estimated using the full covariance matrix. The cell e.s.d.'s are taken into account individually in the estimation of e.s.d.'s in distances, angles and torsion angles; correlations between e.s.d.'s in cell parameters are only used when they are defined by crystal symmetry. An approximate (isotropic) treatment of cell e.s.d.'s is used for estimating e.s.d.'s involving l.s. planes.
Refinement. Refinement of *F*^2^ against ALL reflections. The weighted *R*-factor *wR* and goodness of fit *S* are based on *F*^2^, conventional *R*-factors *R* are based on *F*, with *F* set to zero for negative *F*^2^. The threshold expression of *F*^2^ > σ(*F*^2^) is used only for calculating *R*-factors(gt) *etc*. and is not relevant to the choice of reflections for refinement. *R*-factors based on *F*^2^ are statistically about twice as large as those based on *F*, and *R*- factors based on ALL data will be even larger.

### Fractional atomic coordinates and isotropic or equivalent isotropic displacement parameters (Å^2^)

**Table:**

	*x*	*y*	*z*	*U*_iso_*/*U*_eq_	
C1	0.2859 (2)	0.4608 (3)	0.27681 (13)	0.0522 (8)	
C2	0.2691 (2)	0.3992 (3)	0.33290 (12)	0.0485 (7)	
H2	0.2124	0.4308	0.3460	0.058*	
C3	0.2670 (2)	0.2506 (3)	0.33063 (13)	0.0482 (7)	
C4	0.4313 (2)	0.2457 (3)	0.34865 (14)	0.0484 (7)	
C5	0.4330 (2)	0.3935 (3)	0.35421 (12)	0.0495 (7)	
H5	0.4803	0.4192	0.3812	0.059*	
C6	0.4467 (2)	0.4608 (3)	0.29906 (13)	0.0537 (8)	
C7	0.3440 (2)	0.4355 (3)	0.37191 (12)	0.0505 (8)	
C8	0.2128 (2)	0.4241 (3)	0.23443 (12)	0.0500 (8)	
C9	0.2286 (2)	0.3441 (3)	0.19141 (13)	0.0576 (8)	
H9	0.2865	0.3149	0.1868	0.069*	
C10	0.1592 (2)	0.3064 (3)	0.15477 (14)	0.0627 (9)	
H10	0.1704	0.2531	0.1257	0.075*	
C11	0.0742 (2)	0.3494 (4)	0.16243 (14)	0.0602 (9)	
C12	0.0572 (2)	0.4299 (4)	0.20424 (14)	0.0640 (9)	
H12	−0.0008	0.4592	0.2085	0.077*	
C13	0.1257 (2)	0.4674 (3)	0.23989 (14)	0.0603 (9)	
H13	0.1139	0.5226	0.2682	0.072*	
C14	0.1801 (2)	0.2023 (3)	0.35236 (13)	0.0499 (7)	
C15	0.1040 (2)	0.1871 (3)	0.31863 (14)	0.0596 (9)	
H15	0.1071	0.2026	0.2816	0.072*	
C16	0.0231 (2)	0.1492 (4)	0.33852 (17)	0.0721 (10)	
H16	−0.0278	0.1408	0.3153	0.086*	
C17	0.0193 (2)	0.1245 (4)	0.39275 (18)	0.0756 (11)	
C18	0.0929 (3)	0.1397 (4)	0.42751 (16)	0.0820 (12)	
H18	0.0891	0.1241	0.4645	0.098*	
C19	0.1732 (2)	0.1785 (4)	0.40740 (14)	0.0676 (10)	
H19	0.2233	0.1889	0.4312	0.081*	
C20	0.5042 (2)	0.1854 (3)	0.38518 (13)	0.0513 (8)	
C21	0.4914 (2)	0.1632 (3)	0.43962 (14)	0.0597 (9)	
H21	0.4358	0.1804	0.4534	0.072*	
C22	0.5599 (3)	0.1161 (4)	0.47385 (15)	0.0685 (10)	
H22	0.5511	0.1033	0.5106	0.082*	
C23	0.6403 (2)	0.0886 (4)	0.45328 (17)	0.0688 (10)	
C24	0.6552 (2)	0.1077 (4)	0.39992 (17)	0.0744 (11)	
H24	0.7105	0.0878	0.3865	0.089*	
C25	0.5870 (2)	0.1571 (4)	0.36575 (15)	0.0645 (9)	
H25	0.5972	0.1715	0.3293	0.077*	
C26	0.5374 (2)	0.4319 (3)	0.27753 (13)	0.0559 (8)	
C27	0.6118 (3)	0.4936 (4)	0.29977 (17)	0.0784 (11)	
H27	0.6051	0.5533	0.3271	0.094*	
C28	0.6954 (3)	0.4685 (5)	0.2823 (2)	0.0986 (15)	
H28	0.7454	0.5104	0.2978	0.118*	
C29	0.7049 (3)	0.3807 (5)	0.2415 (2)	0.0927 (14)	
C30	0.6326 (3)	0.3186 (5)	0.21902 (17)	0.0860 (13)	
H30	0.6395	0.2587	0.1917	0.103*	
C31	0.5497 (3)	0.3444 (4)	0.23664 (15)	0.0698 (10)	
H31	0.5001	0.3021	0.2208	0.084*	
C32	0.3717 (4)	0.5788 (5)	0.49734 (18)	0.1062 (16)	
H32A	0.3446	0.6590	0.4858	0.127*	
H32B	0.4242	0.5979	0.5208	0.127*	
C33	0.3056 (4)	0.5049 (6)	0.52925 (17)	0.0944 (14)	
C34	0.2425 (5)	0.5741 (8)	0.5549 (2)	0.136 (2)	
H34	0.2418	0.6624	0.5522	0.163*	
C35	0.1807 (6)	0.5133 (14)	0.5844 (4)	0.194 (5)	
H35	0.1375	0.5612	0.6010	0.233*	
C36	0.1804 (9)	0.3853 (15)	0.5902 (4)	0.198 (6)	
H36	0.1380	0.3457	0.6107	0.238*	
C37	0.2455 (9)	0.3128 (11)	0.5645 (4)	0.198 (5)	
H37	0.2464	0.2245	0.5671	0.238*	
C38	0.3095 (5)	0.3786 (6)	0.5345 (2)	0.1211 (19)	
H38	0.3545	0.3334	0.5183	0.145*	
C39	0.0817 (4)	0.6394 (6)	0.4002 (2)	0.1086 (17)	
C40	0.0896 (7)	0.7529 (8)	0.4329 (3)	0.213 (4)	
H40A	0.0921	0.8260	0.4096	0.320*	
H40B	0.1433	0.7486	0.4561	0.320*	
H40C	0.0385	0.7598	0.4548	0.320*	
C41	0.0762 (4)	0.5122 (7)	0.4283 (3)	0.142 (2)	
H41A	0.0714	0.4458	0.4015	0.213*	
H41B	0.0245	0.5106	0.4498	0.213*	
H41C	0.1293	0.4993	0.4514	0.213*	
N1	0.37419 (18)	0.4222 (3)	0.26073 (11)	0.0534 (7)	
N2	0.34337 (17)	0.2004 (3)	0.36383 (11)	0.0519 (7)	
N3	0.3236 (2)	0.4941 (3)	0.41573 (12)	0.0745 (9)	
O1	0.39933 (19)	0.5102 (3)	0.44974 (12)	0.0930 (9)	
O2	0.0791 (4)	0.6448 (5)	0.35250 (17)	0.1589 (19)	
Cl1	−0.01371 (7)	0.29806 (12)	0.11818 (4)	0.0871 (4)	
Cl2	−0.08182 (8)	0.07631 (17)	0.41840 (6)	0.1228 (5)	
Cl3	0.72648 (8)	0.03096 (14)	0.49745 (6)	0.1128 (5)	
Cl4	0.81177 (10)	0.3490 (2)	0.21892 (8)	0.1549 (7)	
H1	0.286 (2)	0.557 (3)	0.2824 (12)	0.060 (9)*	
H1A	0.386 (2)	0.453 (3)	0.2293 (14)	0.060 (10)*	
H2A	0.343 (2)	0.117 (3)	0.3623 (13)	0.059 (10)*	
H3	0.2687 (19)	0.225 (3)	0.2914 (13)	0.050 (8)*	
H4	0.4428 (18)	0.225 (3)	0.3110 (12)	0.042 (8)*	
H6	0.4450 (19)	0.551 (3)	0.3059 (12)	0.049 (8)*	

### Atomic displacement parameters (Å^2^)

**Table:**

	*U*^11^	*U*^22^	*U*^33^	*U*^12^	*U*^13^	*U*^23^
C1	0.060 (2)	0.0453 (19)	0.0517 (19)	0.0018 (16)	0.0036 (15)	0.0040 (15)
C2	0.0522 (18)	0.0474 (18)	0.0463 (17)	0.0051 (15)	0.0063 (14)	0.0020 (14)
C3	0.0505 (18)	0.0478 (18)	0.0461 (18)	0.0029 (15)	0.0002 (14)	0.0030 (14)
C4	0.0481 (18)	0.0472 (18)	0.0501 (19)	0.0000 (15)	0.0030 (14)	−0.0009 (15)
C5	0.0553 (19)	0.0464 (18)	0.0464 (17)	−0.0041 (15)	−0.0011 (14)	−0.0015 (14)
C6	0.061 (2)	0.048 (2)	0.0523 (19)	−0.0040 (16)	0.0021 (16)	0.0021 (15)
C7	0.066 (2)	0.0440 (18)	0.0410 (17)	0.0014 (15)	0.0035 (15)	0.0010 (14)
C8	0.0557 (19)	0.0471 (18)	0.0470 (17)	0.0019 (15)	0.0010 (14)	0.0087 (14)
C9	0.057 (2)	0.063 (2)	0.0536 (19)	0.0061 (17)	0.0057 (16)	0.0036 (17)
C10	0.068 (2)	0.067 (2)	0.0526 (19)	0.0004 (19)	0.0006 (17)	−0.0006 (17)
C11	0.059 (2)	0.068 (2)	0.052 (2)	−0.0034 (18)	−0.0042 (16)	0.0173 (17)
C12	0.053 (2)	0.074 (2)	0.065 (2)	0.0100 (18)	0.0030 (17)	0.0140 (19)
C13	0.064 (2)	0.061 (2)	0.056 (2)	0.0126 (18)	0.0047 (17)	0.0060 (16)
C14	0.0512 (18)	0.0451 (17)	0.0533 (18)	0.0049 (14)	0.0008 (14)	0.0026 (14)
C15	0.059 (2)	0.063 (2)	0.0567 (19)	0.0010 (17)	−0.0018 (16)	−0.0045 (17)
C16	0.052 (2)	0.080 (3)	0.083 (3)	−0.0030 (19)	−0.0057 (19)	−0.008 (2)
C17	0.052 (2)	0.085 (3)	0.091 (3)	−0.004 (2)	0.011 (2)	0.010 (2)
C18	0.064 (2)	0.114 (4)	0.068 (2)	−0.002 (2)	0.010 (2)	0.029 (2)
C19	0.052 (2)	0.090 (3)	0.060 (2)	−0.0023 (19)	−0.0036 (16)	0.0198 (19)
C20	0.0519 (19)	0.0467 (18)	0.0549 (19)	−0.0043 (15)	−0.0009 (15)	0.0033 (15)
C21	0.056 (2)	0.062 (2)	0.061 (2)	0.0026 (17)	0.0039 (16)	0.0051 (17)
C22	0.069 (2)	0.073 (2)	0.062 (2)	−0.009 (2)	−0.0059 (19)	0.0179 (19)
C23	0.053 (2)	0.067 (2)	0.084 (3)	−0.0034 (18)	−0.0143 (19)	0.022 (2)
C24	0.049 (2)	0.087 (3)	0.086 (3)	0.0041 (19)	−0.0024 (19)	0.008 (2)
C25	0.054 (2)	0.077 (2)	0.063 (2)	0.0012 (18)	0.0042 (17)	0.0065 (18)
C26	0.058 (2)	0.058 (2)	0.0521 (19)	−0.0079 (17)	0.0031 (15)	0.0050 (16)
C27	0.063 (2)	0.082 (3)	0.091 (3)	−0.016 (2)	0.009 (2)	−0.016 (2)
C28	0.066 (3)	0.111 (4)	0.119 (4)	−0.026 (3)	0.008 (3)	−0.014 (3)
C29	0.069 (3)	0.112 (4)	0.100 (3)	−0.008 (3)	0.028 (2)	0.000 (3)
C30	0.080 (3)	0.101 (3)	0.080 (3)	−0.006 (3)	0.023 (2)	−0.022 (2)
C31	0.064 (2)	0.085 (3)	0.060 (2)	−0.010 (2)	0.0076 (18)	−0.010 (2)
C32	0.141 (5)	0.107 (4)	0.071 (3)	−0.015 (3)	0.002 (3)	−0.039 (3)
C33	0.118 (4)	0.111 (4)	0.053 (2)	0.005 (3)	−0.004 (3)	−0.016 (3)
C34	0.138 (5)	0.175 (7)	0.094 (4)	0.021 (5)	0.004 (4)	−0.004 (4)
C35	0.111 (6)	0.358 (17)	0.116 (6)	−0.002 (8)	0.022 (4)	0.019 (9)
C36	0.222 (12)	0.281 (17)	0.090 (6)	−0.079 (11)	−0.007 (6)	0.065 (8)
C37	0.296 (15)	0.192 (10)	0.102 (6)	−0.072 (10)	−0.037 (7)	0.038 (6)
C38	0.171 (6)	0.104 (5)	0.087 (4)	−0.008 (4)	−0.010 (4)	0.011 (3)
C39	0.116 (4)	0.124 (5)	0.086 (4)	0.021 (3)	0.013 (3)	−0.010 (3)
C40	0.314 (12)	0.155 (7)	0.170 (7)	0.005 (7)	−0.010 (7)	−0.062 (6)
C41	0.126 (5)	0.164 (6)	0.138 (5)	0.017 (4)	0.038 (4)	0.031 (5)
N1	0.0511 (16)	0.0626 (18)	0.0465 (16)	−0.0030 (13)	0.0039 (13)	0.0057 (14)
N2	0.0484 (16)	0.0447 (17)	0.0623 (17)	0.0005 (13)	0.0006 (12)	0.0088 (13)
N3	0.090 (2)	0.072 (2)	0.0593 (18)	−0.0117 (18)	−0.0146 (17)	0.0008 (16)
O1	0.0775 (19)	0.116 (2)	0.0848 (19)	−0.0107 (17)	0.0013 (15)	−0.0291 (17)
O2	0.234 (5)	0.156 (4)	0.087 (3)	0.043 (4)	0.015 (3)	0.002 (3)
Cl1	0.0746 (7)	0.1043 (8)	0.0801 (7)	−0.0127 (6)	−0.0181 (5)	0.0094 (6)
Cl2	0.0633 (7)	0.1634 (14)	0.1442 (12)	−0.0164 (8)	0.0286 (7)	0.0234 (10)
Cl3	0.0765 (8)	0.1344 (11)	0.1236 (10)	0.0078 (7)	−0.0308 (7)	0.0487 (8)
Cl4	0.0784 (9)	0.1845 (17)	0.2077 (18)	−0.0109 (10)	0.0618 (10)	−0.0301 (14)

### Geometric parameters (Å, º)

**Table:**

C1—N1	1.454 (4)	C21—H21	0.9300
C1—C8	1.517 (4)	C22—C23	1.358 (5)
C1—C2	1.557 (4)	C22—H22	0.9300
C1—H1	1.02 (3)	C23—C24	1.358 (5)
C2—C7	1.482 (4)	C23—Cl3	1.746 (3)
C2—C3	1.562 (4)	C24—C25	1.385 (5)
C2—H2	0.9800	C24—H24	0.9300
C3—N2	1.465 (4)	C25—H25	0.9300
C3—C14	1.516 (4)	C26—C27	1.373 (5)
C3—H3	1.00 (3)	C26—C31	1.383 (5)
C4—N2	1.463 (4)	C27—C28	1.368 (6)
C4—C20	1.513 (4)	C27—H27	0.9300
C4—C5	1.558 (4)	C28—C29	1.375 (6)
C4—H4	0.98 (3)	C28—H28	0.9300
C5—C7	1.488 (4)	C29—C30	1.354 (6)
C5—C6	1.553 (4)	C29—Cl4	1.750 (4)
C5—H5	0.9800	C30—C31	1.360 (5)
C6—N1	1.454 (4)	C30—H30	0.9300
C6—C26	1.510 (5)	C31—H31	0.9300
C6—H6	0.97 (3)	C32—O1	1.454 (5)
C7—N3	1.292 (4)	C32—C33	1.508 (7)
C8—C9	1.382 (4)	C32—H32A	0.9700
C8—C13	1.391 (4)	C32—H32B	0.9700
C9—C10	1.393 (5)	C33—C38	1.334 (7)
C9—H9	0.9300	C33—C34	1.371 (8)
C10—C11	1.369 (5)	C34—C35	1.363 (11)
C10—H10	0.9300	C34—H34	0.9300
C11—C12	1.366 (5)	C35—C36	1.353 (15)
C11—Cl1	1.744 (4)	C35—H35	0.9300
C12—C13	1.369 (5)	C36—C37	1.410 (14)
C12—H12	0.9300	C36—H36	0.9300
C13—H13	0.9300	C37—C38	1.418 (11)
C14—C15	1.379 (4)	C37—H37	0.9300
C14—C19	1.386 (5)	C38—H38	0.9300
C15—C16	1.386 (5)	C39—O2	1.173 (6)
C15—H15	0.9300	C39—C40	1.441 (9)
C16—C17	1.363 (5)	C39—C41	1.509 (8)
C16—H16	0.9300	C40—H40A	0.9600
C17—C18	1.364 (5)	C40—H40B	0.9600
C17—Cl2	1.743 (4)	C40—H40C	0.9600
C18—C19	1.382 (5)	C41—H41A	0.9600
C18—H18	0.9300	C41—H41B	0.9600
C19—H19	0.9300	C41—H41C	0.9600
C20—C25	1.382 (5)	N1—H1A	0.86 (3)
C20—C21	1.383 (5)	N2—H2A	0.88 (4)
C21—C22	1.381 (5)	N3—O1	1.380 (4)
			
N1—C1—C8	111.7 (3)	C22—C21—H21	119.5
N1—C1—C2	108.5 (3)	C20—C21—H21	119.5
C8—C1—C2	111.0 (3)	C23—C22—C21	119.3 (3)
N1—C1—H1	108.4 (18)	C23—C22—H22	120.4
C8—C1—H1	110.1 (18)	C21—C22—H22	120.4
C2—C1—H1	106.9 (18)	C24—C23—C22	121.5 (3)
C7—C2—C1	108.3 (3)	C24—C23—Cl3	119.9 (3)
C7—C2—C3	107.1 (2)	C22—C23—Cl3	118.5 (3)
C1—C2—C3	112.8 (3)	C23—C24—C25	119.3 (4)
C7—C2—H2	109.5	C23—C24—H24	120.4
C1—C2—H2	109.5	C25—C24—H24	120.4
C3—C2—H2	109.5	C20—C25—C24	120.8 (3)
N2—C3—C14	109.7 (3)	C20—C25—H25	119.6
N2—C3—C2	109.1 (3)	C24—C25—H25	119.6
C14—C3—C2	109.8 (3)	C27—C26—C31	117.8 (3)
N2—C3—H3	112.2 (17)	C27—C26—C6	119.1 (3)
C14—C3—H3	108.4 (17)	C31—C26—C6	123.1 (3)
C2—C3—H3	107.7 (17)	C28—C27—C26	121.1 (4)
N2—C4—C20	109.8 (3)	C28—C27—H27	119.5
N2—C4—C5	108.3 (3)	C26—C27—H27	119.5
C20—C4—C5	110.9 (3)	C27—C28—C29	119.3 (4)
N2—C4—H4	112.1 (17)	C27—C28—H28	120.3
C20—C4—H4	108.2 (16)	C29—C28—H28	120.3
C5—C4—H4	107.6 (17)	C30—C29—C28	120.7 (4)
C7—C5—C6	106.4 (3)	C30—C29—Cl4	119.9 (4)
C7—C5—C4	108.1 (3)	C28—C29—Cl4	119.4 (4)
C6—C5—C4	112.3 (3)	C29—C30—C31	119.5 (4)
C7—C5—H5	110.0	C29—C30—H30	120.3
C6—C5—H5	110.0	C31—C30—H30	120.3
C4—C5—H5	110.0	C30—C31—C26	121.6 (4)
N1—C6—C26	111.5 (3)	C30—C31—H31	119.2
N1—C6—C5	108.1 (3)	C26—C31—H31	119.2
C26—C6—C5	112.2 (3)	O1—C32—C33	112.7 (4)
N1—C6—H6	111.3 (18)	O1—C32—H32A	109.0
C26—C6—H6	106.9 (18)	C33—C32—H32A	109.0
C5—C6—H6	106.8 (18)	O1—C32—H32B	109.0
N3—C7—C2	117.4 (3)	C33—C32—H32B	109.0
N3—C7—C5	129.8 (3)	H32A—C32—H32B	107.8
C2—C7—C5	112.8 (3)	C38—C33—C34	120.7 (6)
C9—C8—C13	118.1 (3)	C38—C33—C32	122.4 (6)
C9—C8—C1	122.4 (3)	C34—C33—C32	116.8 (6)
C13—C8—C1	119.4 (3)	C35—C34—C33	119.9 (9)
C8—C9—C10	121.2 (3)	C35—C34—H34	120.1
C8—C9—H9	119.4	C33—C34—H34	120.1
C10—C9—H9	119.4	C36—C35—C34	121.9 (11)
C11—C10—C9	118.6 (3)	C36—C35—H35	119.1
C11—C10—H10	120.7	C34—C35—H35	119.1
C9—C10—H10	120.7	C35—C36—C37	118.9 (12)
C12—C11—C10	121.3 (3)	C35—C36—H36	120.5
C12—C11—Cl1	119.8 (3)	C37—C36—H36	120.5
C10—C11—Cl1	118.9 (3)	C36—C37—C38	118.0 (11)
C11—C12—C13	119.8 (3)	C36—C37—H37	121.0
C11—C12—H12	120.1	C38—C37—H37	121.0
C13—C12—H12	120.1	C33—C38—C37	120.6 (8)
C12—C13—C8	120.9 (3)	C33—C38—H38	119.7
C12—C13—H13	119.5	C37—C38—H38	119.7
C8—C13—H13	119.5	O2—C39—C40	121.1 (7)
C15—C14—C19	117.6 (3)	O2—C39—C41	120.1 (6)
C15—C14—C3	121.2 (3)	C40—C39—C41	118.8 (6)
C19—C14—C3	121.1 (3)	C39—C40—H40A	109.5
C14—C15—C16	121.7 (3)	C39—C40—H40B	109.5
C14—C15—H15	119.1	H40A—C40—H40B	109.5
C16—C15—H15	119.1	C39—C40—H40C	109.5
C17—C16—C15	118.9 (3)	H40A—C40—H40C	109.5
C17—C16—H16	120.5	H40B—C40—H40C	109.5
C15—C16—H16	120.5	C39—C41—H41A	109.5
C16—C17—C18	121.1 (4)	C39—C41—H41B	109.5
C16—C17—Cl2	119.5 (3)	H41A—C41—H41B	109.5
C18—C17—Cl2	119.4 (3)	C39—C41—H41C	109.5
C17—C18—C19	119.6 (4)	H41A—C41—H41C	109.5
C17—C18—H18	120.2	H41B—C41—H41C	109.5
C19—C18—H18	120.2	C1—N1—C6	113.5 (3)
C18—C19—C14	121.0 (3)	C1—N1—H1A	112 (2)
C18—C19—H19	119.5	C6—N1—H1A	107 (2)
C14—C19—H19	119.5	C4—N2—C3	114.9 (3)
C25—C20—C21	118.2 (3)	C4—N2—H2A	109 (2)
C25—C20—C4	120.9 (3)	C3—N2—H2A	109 (2)
C21—C20—C4	120.8 (3)	C7—N3—O1	110.0 (3)
C22—C21—C20	120.9 (3)	N3—O1—C32	106.9 (3)
			
N1—C1—C2—C7	55.2 (3)	C3—C14—C19—C18	177.2 (4)
C8—C1—C2—C7	178.4 (3)	N2—C4—C20—C25	146.8 (3)
N1—C1—C2—C3	−63.2 (3)	C5—C4—C20—C25	−93.6 (4)
C8—C1—C2—C3	60.0 (3)	N2—C4—C20—C21	−36.6 (4)
C7—C2—C3—N2	−4.7 (3)	C5—C4—C20—C21	83.0 (4)
C1—C2—C3—N2	114.4 (3)	C25—C20—C21—C22	1.0 (5)
C7—C2—C3—C14	115.5 (3)	C4—C20—C21—C22	−175.6 (3)
C1—C2—C3—C14	−125.4 (3)	C20—C21—C22—C23	−1.5 (6)
N2—C4—C5—C7	−1.1 (3)	C21—C22—C23—C24	0.7 (6)
C20—C4—C5—C7	−121.6 (3)	C21—C22—C23—Cl3	179.2 (3)
N2—C4—C5—C6	−118.2 (3)	C22—C23—C24—C25	0.4 (6)
C20—C4—C5—C6	121.4 (3)	Cl3—C23—C24—C25	−178.0 (3)
C7—C5—C6—N1	−59.7 (3)	C21—C20—C25—C24	0.1 (5)
C4—C5—C6—N1	58.4 (3)	C4—C20—C25—C24	176.8 (3)
C7—C5—C6—C26	177.0 (3)	C23—C24—C25—C20	−0.9 (6)
C4—C5—C6—C26	−65.0 (4)	N1—C6—C26—C27	161.1 (3)
C1—C2—C7—N3	122.5 (3)	C5—C6—C26—C27	−77.5 (4)
C3—C2—C7—N3	−115.5 (3)	N1—C6—C26—C31	−19.9 (5)
C1—C2—C7—C5	−59.2 (3)	C5—C6—C26—C31	101.6 (4)
C3—C2—C7—C5	62.7 (3)	C31—C26—C27—C28	−0.5 (6)
C6—C5—C7—N3	−121.0 (4)	C6—C26—C27—C28	178.7 (4)
C4—C5—C7—N3	118.3 (4)	C26—C27—C28—C29	0.5 (7)
C6—C5—C7—C2	61.1 (3)	C27—C28—C29—C30	−0.5 (8)
C4—C5—C7—C2	−59.7 (3)	C27—C28—C29—Cl4	179.7 (4)
N1—C1—C8—C9	10.8 (4)	C28—C29—C30—C31	0.6 (8)
C2—C1—C8—C9	−110.5 (3)	Cl4—C29—C30—C31	−179.7 (4)
N1—C1—C8—C13	−172.1 (3)	C29—C30—C31—C26	−0.7 (7)
C2—C1—C8—C13	66.6 (4)	C27—C26—C31—C30	0.6 (6)
C13—C8—C9—C10	−0.7 (5)	C6—C26—C31—C30	−178.5 (4)
C1—C8—C9—C10	176.4 (3)	O1—C32—C33—C38	35.3 (7)
C8—C9—C10—C11	−0.6 (5)	O1—C32—C33—C34	−147.2 (5)
C9—C10—C11—C12	1.5 (5)	C38—C33—C34—C35	−2.5 (9)
C9—C10—C11—Cl1	−177.7 (3)	C32—C33—C34—C35	179.9 (6)
C10—C11—C12—C13	−1.0 (5)	C33—C34—C35—C36	1.2 (13)
Cl1—C11—C12—C13	178.2 (3)	C34—C35—C36—C37	−0.6 (16)
C11—C12—C13—C8	−0.4 (5)	C35—C36—C37—C38	1.1 (15)
C9—C8—C13—C12	1.2 (5)	C34—C33—C38—C37	3.2 (9)
C1—C8—C13—C12	−176.0 (3)	C32—C33—C38—C37	−179.4 (5)
N2—C3—C14—C15	−151.8 (3)	C36—C37—C38—C33	−2.5 (12)
C2—C3—C14—C15	88.3 (4)	C8—C1—N1—C6	177.3 (3)
N2—C3—C14—C19	31.7 (4)	C2—C1—N1—C6	−60.0 (3)
C2—C3—C14—C19	−88.1 (4)	C26—C6—N1—C1	−173.5 (3)
C19—C14—C15—C16	−0.1 (5)	C5—C6—N1—C1	62.8 (4)
C3—C14—C15—C16	−176.7 (3)	C20—C4—N2—C3	−179.0 (3)
C14—C15—C16—C17	−1.1 (6)	C5—C4—N2—C3	59.8 (3)
C15—C16—C17—C18	1.7 (6)	C14—C3—N2—C4	−176.9 (3)
C15—C16—C17—Cl2	−179.8 (3)	C2—C3—N2—C4	−56.6 (4)
C16—C17—C18—C19	−1.2 (7)	C2—C7—N3—O1	174.4 (3)
Cl2—C17—C18—C19	−179.7 (3)	C5—C7—N3—O1	−3.5 (5)
C17—C18—C19—C14	0.0 (7)	C7—N3—O1—C32	178.8 (3)
C15—C14—C19—C18	0.7 (6)	C33—C32—O1—N3	63.3 (5)

### Hydrogen-bond geometry (Å, º)

Cg3 is the centroid of the C8–C13 ring.

**Table:**

*D*—H···*A*	*D*—H	H···*A*	*D*···*A*	*D*—H···*A*
C13—H13···O2	0.93	2.52	3.441 (6)	172
N2—H2*A*···*Cg*3^i^	0.88 (3)	2.85 (3)	3.637 (3)	150 (3)

Symmetry code: (i) −*x*+1/2, *y*+3/2, −*z*+1/2.

## References

[bb12] Altomare, A., Cascarano, G., Giacovazzo, C. & Guagliardi, A. (1993). *J. Appl. Cryst.* **26**, 343–350.

[bb1] Asakawa, Y. (1995). *In Progress in the Chemistry of Organic Natural Products*, edited by G. W. Moore, R. E. Steglich & W. Tamm. New York: Springer-Verlag.

[bb2] Bruker (2004). *APEX2*, *SAINT-Plus* and *SADABS* Bruker AXS Inc., Madison, Wisconsin, USA.

[bb3] Cremer, D. & Pople, J. A. (1975). *J. Am. Chem. Soc.* **97**, 1354–1358.

[bb4] Farrugia, L. J. (1997). *J. Appl. Cryst.* **30**, 565.

[bb5] Jayaraman, R. & Avila, S. (1981). *Chem. Rev.* **81**, 149–174.

[bb6] Luger, P. & Bülow, R. (1983). *J. Appl. Cryst.* **16**, 431–432.

[bb7] Nardelli, M. (1983). *Acta Cryst.* C**39**, 1141–1142.

[bb8] Parthiban, P., Aridoss, G., Rathika, P., Ramkumar, V. & Kabilan, S. (2009). *Bioorg. Med. Chem. Lett.* **19**, 6981–6985. 10.1016/j.bmcl.2009.10.042 19879756

[bb9] Parthiban, P., Kabilan, S., Ramkumar, V. & Jeong, Y. T. (2010). *Bioorg. Med. Chem. Lett* **20**, 6452–6458. 10.1016/j.bmcl.2010.09.079 20933407

[bb10] Parthiban, P., Ramachandran, R., Aridoss, G. & Kabilan, S. (2008). *Magn. Reson. Chem* **46**, 780–785. 10.1002/mrc.2243 18509862

[bb11] Sheldrick, G. M. (2008). *Acta Cryst.* A**64**, 112–122. 10.1107/S0108767307043930 18156677

